# Comparative analysis of the influence of Chinese urban and rural family cultures on household financial asset allocation

**DOI:** 10.3389/fpsyg.2023.1119258

**Published:** 2023-02-08

**Authors:** Zhisheng Li, Cucci Mirko, Maria Teresa D’Agostino, Jiyang Jin

**Affiliations:** ^1^School of Business, Hunan Agricultural University, Changsha, China; ^2^Department of Computer Sciences, University of Turin, Turin, Italy; ^3^Department of Economics and Law, University of Cassino, Cassino, Italy; ^4^College of Innovative Business and Accountancy, Dhurakij Pundit University, Bangkok, Thailand

**Keywords:** family culture, family cultural values, cultural psychology, Hofstede’s cultural values, cognition, household financial asset allocation, knowledge acquisition

## Abstract

Despite the national strategic priority to achieve common prosperity, there still exist prominent discrepancies in financial asset allocation between Chinese urban and rural families, which requires a deeper, more comprehensive investigation. To fill this gap, the present research adopted a cultural perspective to investigate relevant issues by addressing the cognitive differences of residents between urban and rural families. Under the analytical framework of Hofstede’s cultural values, this paper discusses the cognitive differences between urban and rural families in terms of financial asset allocation in the cultural dimensions of collectivism, individualism and uncertainty avoidance; hypotheses are accordingly developed. In terms of research methods, the data of the China Family Panel Studies (CFPS) were used in the probit model to investigate the influence of urban and rural family cultural differences on household financial asset allocation. The results of this paper are as follows: (1) family cultural values have a positive impact on family financial asset allocation; (2) knowledge acquisition plays an intermediary role in family cultural values and family financial asset allocation; (3) and for rural families with high collectivism and uncertainty avoidance, the mediating effect is more prominent. This paper provides a new perspective for exploring the possibility of household asset allocation from the perspective of cultural psychology. The contribution of this paper have theoretical and practical reference significance to narrow the wealth gap between urban and rural areas and achieve common prosperity.

## 1. Introduction

As two distinct geographical regions, urban and rural areas have cultural and psychological differences in the process of development and integration. The accelerated integration of urban and rural areas has, to some extent, promoted the exchange and integration of urban and rural culture, but the long-term cultural psychology (Chentsova-Dutton and Maercker, 2019; Mo et al., 2022) of urban and rural residents has also shaped the varied cognition and perception of urban and rural residents. Therefore, significant differences remain in the value system and ways of thinking, inevitably affecting the cultural values of both urban and rural areas. The differences in cultural values between urban and rural areas are an inevitable result of historical development; furthermore, these differences form the basis of certain stereotypes of urban and rural residents, which is not conducive to achieving common prosperity.

In the context of the recent promotion of common prosperity in China, the gap between the rich and the poor remains significant, hindering the overall consumption demand and detrimental to the transformation and upgrading of the existing economic structure. The total global wealth up to the end of 2021 has been estimated at 463.6 trillion US dollars,^1^ an increase of 9.8% over the previous year and far higher than the average annual increase of 6.6% since the beginning of this century. Meanwhile, the scale of Chinese household wealth has also continued to grow. In 2019, the total household wealth in China was 78.08 trillion dollars, a 21-fold increase over 3.7 trillion in 2000, now ranking second in the world. With the rapid development of financial markets and the increase in family wealth, the importance of family finance has become increasingly prominent. As an indispensable part of the national economy, family plays an important role in society, making family finance a research hotspot. According to the data of China Family Panel Studies (CFPS), the proportion of financial products owned by Chinese residents in 2020 was 6.13%, including 11.16% in urban areas and 1.84% in rural areas.^2^ Thus, there is clearly a large gap between urban and rural household financial asset allocation. Furthermore, the overall household financial asset allocation rate in China has not increased significantly, even showing a slight downward trend. The unbalanced limited participation of urban and rural areas becomes an extremely urgent topic in current research.

As a typical representative of rural encircle cities, China vigorously promotes the strategy of common prosperity, and the integrated development of regional culture and psychology is increasinglly important (Chin et al., 2022a). For example, the largest problem of urban and rural cultural exchange is reflected in the differences in terms of cultural psychology. Therefore, to promote the common development of urban and rural areas, the cultural psychology of urban and rural families should be taken as the entry point for research, thus facilitating the analysis of differences in family cultural values and cognition within these two cultural backgrounds (Li, 2007). Previous studies have analyzed the gap between urban and rural households’ financial asset allocation from the perspective of financial technology (Campanella et al., 2020) and macro-economy. The study showed that in China, provinces located in coastal areas like Zhejiang and Shanghai have had frequent commercial exchanges through international business, enhancing local residents’ commercial awareness. Families in such areas have attached great importance to financial investment, while regions in remote western areas like Daliang Mountain in Sichuan Province have been characterized by high mountains, long roads, and a “backward” economy. Residents in the areas like the latter largely make a living *via* agriculture, and their participation in financial asset allocation activities is extremely limited (Shackle and Duesenberry, 1951; Rewilak, 2013). Some scholars have also conducted research on the allocation of household financial assets from micro-perspectives such as household economic situations and the individual characteristics of members. For example, the more wealth a family has, the more willing that family is to participate in financial market investment (Jia et al., 2022; Mulat, 2022). Moreover, family size and annual income affect the choice of financial assets (Donkers et al., 2001; Yavas and Malladi, 2020). The age, investment experience, and financial literacy of a family’s financial decision-maker also have a significant impact on household financial allocation (Bonsang and Dohmen, 2015; Calcagno and Monticone, 2015). A higher education level and better health have been to shown to have significant positive impacts on the allocation of household financial assets (Guiso et al., 2002; Rosen et al., 2003). Stable income is the basic guarantee for financial market investment, and families with stable incomes are more willing to participate in financial asset allocation (Singh and Mohan, 2020). Male family members have been shown to be more will to bear the risks brought by financial allocation than women, with women showing more caution than men (Filippin and Crosetto, 2016; Nelson, 2016).

It is worth noting that studies have verified the impact of the macro- or micro-economy on levels of household financial awareness, resulting in significant differences in asset allocation among families in different regions (Kramer, 2016), as well as differences in overall financial planning (Fligstein and Goldstein, 2015). However, few scholars have explored the impact mechanism of cultural psychology and value differences on household financial asset allocation.

Therefore, in order to address the relevant research gaps, this paper focuses on exploring whether the differences in cultural psychology and value differences between urban and rural families affect their financial asset allocation. The paper also investigates the impact of such differences in urban and rural families. From the perspective of Hofstede’s cultural theory, this paper discusses the possible impact of the differences in urban and rural family cultural psychology and value differences on their financial asset allocation, using CFPS data and the probit model to test the proposed hypotheses. This study is arranged as follows: the second part contains the hypotheses, the third part outlines the methodology, the fourth part presents the empirical analysis, and the fifth part includes the discussion and conclusion.

## 2. Hypotheses

Culture is the basic element of economic development. Cultural factors and economic motivation affect people’s behavior to varying degrees, with people sometimes restricted by cultural factors in their daily economic activities (Marshall, 1981; Chin et al., 2022b). According to Hofstede’s theory, culture refers to the shared values of a group, with people in the same region thinking and acting in a similar way (Beugelsdijk and Welzel, 2018). Studying urban and rural culture from the perspective of cross-cultural psychology has a certain realistic basis and related background. The study of urban and rural cross-cultural psychology in China is a comparative study of the city and the countryside as two typical regions (Chin et al., 2022c). The city and the countryside are affected by different cultural psychology and have different cultural psychological attributes. Rural culture is deeply rooted in the local, with a strong psychological atmosphere of traditional Chinese culture, while the city culture is affected by the western modern civilization, showing its own unique style. The differences between urban and rural residents in terms of geographical environment, social and economic environment, and cultural values lead to differences in residents’ cognitive psychology, knowledge perception, innovation, and entrepreneurship (Paoloni et al., 2020; Caputo et al., 2021; Yang et al., 2022), ultimately leading to differences in family cultural values, financial knowledge acquisition, and family financial asset selection, among other aspects.

### 2.1. Family cultural values and the allocation of household financial asset

There is a close relationship between cultural values and economic development. Cultural values affect people’s cognition, expectations, and preferences; at the same time, cognition, expectations, and preferences also affect people’s economic behaviors (Fernández and Fogli, 2009; Mostafiz et al., 2021). Grindrod was the first to use cultural values to analyze the impacts of culture on economic development. His study found that individuals had a negative impact on economic development due to the value concept of “amoral familism,” which excessively pursued personal interests, and thus resulted in the imbalance of regional development (Grindrod, 1959). Culture significantly affects people’s investment strategies in information exchange and investment transactions (Fiske, 2003). Stulz and Williamson used religion and language as cultural indicators to test the impact of culture on financial development and found that culture plays a significant role in promoting financial development (Ren et al., 2002). Family is an important and basic unit for financial decision-making (Campbell, 2006; Li et al., 2020), and family cultural values are the direct cause of unbalanced limited participation. Thus, it is necessary to embed cultural values in the discussion of the allocation of household financial assets. Traditional economics suggest that family investors should more clearly understand the spiritual benefits, resource benefits, and asset investment benefits and that cultural values will affect the allocation of household financial assets. However, behavioral economics indicates that family investors’ harmonious cultural values are affected to varying degrees by different policies, systems, and social interactions in their environments; thus, the allocation of household financial assets will also show some differences (He et al., 2016). Therefore, family cultural values will impact their participation in financial market investment. Based on this, we proposed the following hypothesis:

*H1:* Family cultural values have a positive impact on the allocation of household financial assets.

### 2.2. The mediating role of knowledge acquisition

Cultural values lead to differences in beliefs, moral standards, behavior patterns, and other aspects of different ethnic groups, regions, and families, thus producing complex effects on the investment exchanges and economic behaviors of family residents (Zang et al., 2019; Zhou and Cui, 2019). Comprised of long-held traditional historical and cultural symbolism, financial cultures have exerted significant and far-reaching influence on social economies to promote continuous development (Becker and Mulligan, 1997; Smith and Kaminishi, 2020). The allocation of household financial assets represents a financial market investment based on specific cultural values (Lusardi et al., 2017). Family cultural values continuously improve the level of individuals’ financial knowledge through learning by doing in financial practice (Hong et al., 2004; Oanea and Dornean, 2012). In other words, cultural values have an impact on household financial asset allocation through the mediating role of financial knowledge acquisition. Based on learned financial knowledge, family members can make more reasonable decisions in terms of wealth accumulation, financial planning, debt planning, investment management, etc., with such increased financial knowledge perhaps further promoting the allocation of the family’s financial assets. Based on this, we thus propose the following hypothesis:

*H2:* Knowledge acquisition mediates the relationship between the allocation of household financial assets and family cultural values.

### 2.3. Comparison of urban and rural household financial asset allocation

Considering the influence of climate conditions, geographical locations and so on, there are obvious differences between urban and rural families in terms of lifestyle, customs, ideas, and beliefs. Differences in ways of thinking, moral standards, and codes of conduct represented by cultural values have differing impacts on allocation intention and the scale of household financial assets. Therefore, it is necessary to conduct a comparative study between urban and rural areas to examine the influence of culture. Hofstede derived the famous cultural dimension theory from attitudes and values, dividing them into five dimensions according to the cultural differences of different groups, regions, and countries. The five dimensions are as follows: power distance; individualism and collectivism; masculinity and femininity; uncertainty avoidance; and long- and short-term orientation. The allocation of family financial assets is mainly affected by the preference and choice of a family’s financial decision-maker and the family economic situation (Antoniou et al., 2015; Post and Hanewald, 2013). Therefore, for this paper, we selected two dimensions of Hofstede’s five cultural dimensions—namely, individualism and collectivism; and uncertainty avoidance.

Individualism and collectivism reflect the degree of association between individuals and collectives (Ford et al., 2015; Hofstede, 2015). Individualism focuses on individual goals; on the contrary, collectivism emphasizes collective goals. Uncertainty avoidance can be defined as the extent to which the members of a culture feel threatened by uncertain or unknown situations and try to avoid such situations (Hofstede, 1991). Family cultural values can promote the exchange and sharing of financial knowledge. Due to the differences in the average education level and living environment between urban and rural families, the strong financial culture of the city promotes families to acquire more financial knowledge, have reasonable control over the scale of family financial asset allocation, and improve relevant information through mutual communication and sharing. On the other hand, rural families have relatively few channels to obtain financial knowledge, and thus their reasonable ability to control household financial asset allocation is relatively low. In terms of the dimension of individualism and collectivism, the collectivism of rural families is higher than that of urban families. The important financial decision-making behavior often involves the joint participation of rural family members; thus, the knowledge acquisition of rural families has a more profound impact on the allocation of household financial assets. In terms of the dimension of uncertainty avoidance, the higher the degree of uncertainty avoidance is, the lower people’s tolerance for future risks. In contrast, the stronger people’s sense of security is, the higher their tolerance for future risks. The degree of uncertainty avoidance of households in rural areas is relatively high, and the residents are largely conservative and risk averse; as well, the intention and scale of household financial asset allocation are relatively low. Therefore, knowledge acquisition has a more significant impact on a rural family’s allocation intention and scale of financial assets. Hence, we proposed the following:

*H3:* Compared with urban families, the mediating role of knowledge acquisition is more significant in rural families with higher collectivism and a higher degree of uncertainty avoidance.

**Figure 1:**
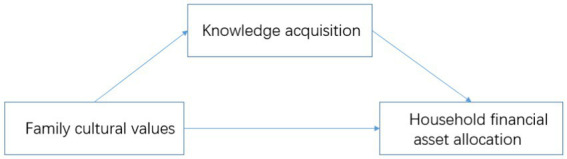
Research framework.

In sum, the influencing mechanism of family cultural values and knowledge acquisition on family financial asset allocation is shown in Figure 1.

## 3. Methodology

### 3.1. Model design

Considering the dependent variable of household financial asset allocation as a dummy variable, we used the probit model to conduct our research. Family financial culture is subordinate to social culture, the latter referring to the investment choices, values, and moral standards of Chinese families under the influence of thousands of years of traditional Confucian cultures. Family participation in the financial market is an investment behavior based on spiritual culture and concept, and thus family financial cultures will impact families’ participation in financial market investment. Family financial culture is the further simplification of the social culture of family participation in financial market investment, and it is also the financial spirit and investment concept of a family’s main investors. Therefore, the independent variable of family cultural values was measured in terms of family financial cultures.

In order to test the impact of family cultural values on household financial asset allocation, the following empirical probit model is constructed in this paper:


(1) $$\text{Prob}\left({\text{Fin}}_{\text{ij}}=1\right)=\Phi \left(\alpha_{0}+\alpha_{1}\;{\text{Cul}}_{\text{ij}}+B_{1}\;{\text{contr}}_{\text{ij}}+\theta \right)$$

Meanwhile, in order to test the mediating effect of financial knowledge acquisition, the following model is constructed:


(2) $$\text{Prob}\left({\text{FK}}_{\text{ij}}=1\right)=\Phi \left(\beta_{0}+\beta_{1}\;{\text{Cul}}_{\text{ij}}+B_{2}\;{\text{contr}}_{\text{ij}}+\theta \right)$$

(3) $$\text{Prob}\left({\text{Fin}}_{\text{ij}}=1\right)=\Phi \left(\lambda_{0}+\lambda_{1}\;{\text{FK}}_{\text{ij}}+\lambda_{2}\;{\text{Cul}}_{\text{ij}}+B_{3}\;{\text{contr}}_{\text{ij}}+\theta \right)$$

Among them, Fin_ij_ represents the variable of household financial asset allocation, Cul_ij_ is the measurement variable of family cultural values, FK_ij_ is the measurement variable of financial knowledge acquisition, contr_ij_ is the control variable, and *θ* represents the random error term. B_1_, B_2_, and B_3_ are the coefficient vectors of the control variables in each corresponding model; *α*_0_, *β*_0_ and *λ*_0_ are the constant terms of each model; *α*_1_, *β*_1_, *λ*_1_, and *λ*_2_ are the coefficients of the explanatory variables in each corresponding model.

### 3.2. Data sources

The data used in this paper are from the China Family Panel Studies (CFPS), which is a national comprehensive social tracking survey project conducted by the China Social Science Survey Center of Peking University. The data is nationwide, extensive, and representative, comprising an important data source for academic and policy research. The database includes community, family, and individual questionnaires. The research objects of this paper are urban and rural families, and the survey objects include all the members of the sample families. The data used in this study were mainly family and personal questionnaire information. Personal information regarding the household head based on family questionnaires includes financial knowledge level, education level, frequency of using Internet learning, gender, age, health status, marital status, etc. Family information based on the family questionnaires includes whether the family holds financial products, family savings, parents’ level of financial knowledge, family size, family income, the proportion of the elderly, the proportion of children, etc.

Since the latest data on household levels in 2020 has not been fully released, this paper utilizes the 2018 CFPS data. Variables of “financial knowledge level” and “parental financial knowledge level” match the 2014 CFPS data in the 2018 CFPS data according to each respondent’s family serial number and the respondent’s serial number. After removing the missing values, duplicate values, outliers, and other unique variables in the sample, 6,623 effective samples were finally obtained, among which 3,334 were urban and 3,289 were rural. The numbers of urban and rural samples in this effective sample were similar, showing good representability.

### 3.3. Key variables

#### 3.3.1. Dependent variable

Household financial asset allocation. Household financial asset allocation is the choice and collocation of household financial assets according to their own economic conditions and the real financial environment in order to increase property income. This variable is directly based on questions such as “Does your family hold financial products, such as stocks, funds, national bonds, trust products, foreign exchange products, etc.?” or “What is the range of your family’s total cash and deposit?” to define the household financial asset allocation, taking the mean value of the variables to measure the household financial asset allocation.

#### 3.3.2. Independent variable

Family cultural values. The variable of family cultural values is measured by family financial culture, which is defined according to the “financial knowledge level” and “parents’ financial knowledge level” in the questionnaire. The main reason is that parents’ words and deeds have a significant influence on their offspring. Therefore, it is of considerable research value to define the family financial culture in terms of the financial knowledge level of the head of the household and the financial knowledge level of the parents, taking the mean value of the variables to measure the family cultural values.

#### 3.3.3. Intermediate variable

Knowledge acquisition. The head of a household, as the head of a family, plays a decisive role in the decision of household financial asset allocation. The personal education level of the household head can represent the initial endowment of knowledge acquisition. Through Internet learning, the household head can acquire relevant knowledge of financial products as well as knowledge about risks and returns, thereby grasping the trends of the financial market at any given time so as to further promote the personal acquisition of the financial knowledge of the household head as well as promote the improvement of the initiative of financial asset allocation driven by internal causes. Knowledge acquisition was defined according to “education level” and “frequency (times) of Internet learning” in the questionnaire, and the mean value of the two variables was taken to measure knowledge acquisition.

#### 3.3.4. Control variable

Other variables affecting household financial asset allocation, family cultural values, and knowledge acquisition were derived from individual questionnaires and family questionnaires. Among them, the variables of gender, age, health status, and marital status at the level of personal characteristics of the household head came from the personal questionnaire; the variables of family size, family income, proportion of the elderly, and proportion of children at the level of family characteristics come from the family questionnaire (Table 1).

**Table 1:** Descriptive statistics (*N* = 6,623).

Variable attribute	Variable dimension	Variables	Method of calculation	Mean	SD
Dependent variable	Household financial asset allocation	Whether hold financial products or not	“YES” = 1, “NO” = 0	0.556	0.323
		Total cash and deposits range	“≥50,000 RMB” = 1, “≤50,000 RMB” = 0	0.794	0.236
Independent variable	Cultural values	Family financial cultures	“The financial knowledge level of the household head is equal to the average level of peers or above” = 1, Otherwise = 0	0.149	0.334
Intermediate variable	Knowledge acquisition	Level of education	“Education degree above college” = 1 Otherwise = 0	0.192	0.394
		Frequency of Internet learning	“1–2 times a week or more” = 1, Otherwise = 0	0.627	0.488
Control variable	Householders’ personal characteristics	Gender	“Male” = 1, “female” = 0	0.493	0.500
		Age	numeric variable/ logarithm	3.864	0.314
		Fitness	“well” = 1, otherwise = 0	0.839	0.387
		Marriage	“Married” = 1, otherwise = 0	0.813	0.371
	Family characteristics	Household size	numeric variable/logarithm	4.347	1.916
		Household income	numeric variable/logarithm	10.706	1.163
		Proportion of elderly	Number of old people/total household population	0.233	0.348
		Proportion of children	Number of children/total household population	0.106	0.307

## 4. Empirical analysis

### 4.1. Regression analysis of family cultural values on household financial asset allocation

Based on the 2018 CFPS data, this paper uses a probit model (1) to estimate the total effects of family cultural values on household financial asset allocation so as to determine whether the conditions are sufficient for further testing of the intermediary effects. The results are shown in Table 2, which reports the impact of family cultural values on household financial asset allocation. Column (a) only considers the univariate relationship between family cultural values and household financial asset allocation, and column (b) and (c) are the estimated results after adding control variables at the personal level of household head and family level. As shown, the influence coefficient of family cultural values in columns (a) to (c) was positive and significant (significant at 1% level), indicating that family cultural values can have a positive effect on financial asset allocation, and the estimated results were consistent with hypothesis H1.

**Table 2:** Regression results of family cultural values on household financial asset allocation.

Household financial asset allocation	(a)	(b)	(c)
Family cultural values	0.723*** (19.45)	0.544*** (9.21)	0.376*** (8.45)
Gender		0.207 (1.40)	0.134* (2.53)
Age		−0.313** (−4.86)	−0.104 (−0.94)
Fitness		0.316*** (4.44)	0.296*** (3.00)
Marriage		0.148*** (8.51)	0.098*** (3.28)
Household size			0.421*** (5.97)
Household income			0.351*** (18.55)
Proportion of elderly			−0.153* (−5.74)
Proportion of children			0.326 (0.82)
*N*	6,623	6,623	6,623
*R* ^2^	0.163	0.172	0.182
Adjusted *R*^2^	0.160	0.169	0.180
*F*	34.116	16.258	10.561

* *p* < 0.05.

** *p* < 0.01.

*** *p* < 0.001.

Further analysis showed that both the personal characteristics of the household head and the family characteristics have an impact on household financial allocation. From the perspective of the characteristic variables of an individual head of household, health level and marital status had a significant positive impact on financial asset allocation. From the perspective of age and the proportion of the elderly, increase in age was shown to hinder the allocation of financial assets. From the perspective of household variables, household income had a significant positive effect on financial asset allocation, and family size had a significant positive correlation with household financial asset allocation.

### 4.2. Analysis of the mediating effect of knowledge acquisition

The comprehensive effects of family cultural values on household financial asset allocation have been previously tested, but the mechanism has not been explained. This study indicates that the promoting effect of family cultural values on household financial asset allocation is mediated by the acquisition of financial knowledge, but whether family cultural values have an effect on household financial asset allocation through the acquisition of financial knowledge as an intermediary needs to be tested. Therefore, family cultural values (Cul) and financial knowledge acquisition (FK) were substituted into Model (2) and Model (3) for regression estimation, and the results are shown in Table 3. Table 3 verifies the mediating effect of financial knowledge acquisition. The regression results showed that the mediating effect of financial knowledge acquisition passed the significance test at the 1% level, and the estimated results were consistent with hypothesis H2, indicating that the influence of family cultural values on household financial asset allocation and financial knowledge acquisition has an intermediary effect.

**Table 3:** Mediating effect of knowledge acquisition.

Variables	Urban areas		Rural areas	
	Knowledge acquisition	Household financial asset allocation	Knowledge acquisition	Household financial asset allocation
Knowledge acquisition		0.166*** (5.86)		0.321*** (5.86)
Family cultural values	0.547*** (3.86)	0.336*** (7.62)	0.465*** (4.77)	0.375*** (6.18)
Gender	0.335* (4.76)	0.164 (2.74)	0.156 (1.38)	−0.113 (−0.96)
Age	−0.337*** (−6.48)	−0.217 (−1.51)	−0.244* (−6.08)	0.195 (1.31)
Fitness	0.206*** (5.42)	0.144** (2.32)	0.219*** (5.67)	0.161** (2.36)
Marriage	−0.066 (−0.92)	0.175*** (4.40)	−0.151 (−0.77)	0.126 (0.34)
Household size	0.389*** (4.55)	0.424*** (6.15)	0.367*** (8.27)	0.372*** (3.63)
Household income	0.486** (2.29)	0.322*** (11.73)	0.198*** (2.15)	0.472*** (13.23)
Proportion of elderly	−0.436 (−8.19)	−0.296* (−5.85)	−0.414 (−8.83)	−0.376 (−2.10)
Proportion of children	−0.217 (−0.64)	−0.055 (−0.01)	0.164 (0.34)	0.195 (0.32)
*N*	3,334	3,334	3,289	3,289
*R* ^2^	0.206	0.205	0.166	0.168
Adjusted *R*^2^	0.204	0.203	0.163	0.162
*F*	8.022	7.559	6.992	6.552

* *p* < 0.05.

** *p* < 0.01.

*** *p* < 0.001.

Further analysis showed that there was a significant negative correlation between age and financial knowledge acquisition, indicating that the older respondents has less need for financial knowledge acquisition. Meanwhile, health level was positively correlated with the acquisition of financial knowledge, indicating that the higher the health level is, the greater the demand for the acquisition of financial knowledge. From the perspective of characteristic variables at the family level, the increase of family size significantly promoted the acquisition of financial knowledge, indicating that the larger the family size is, the more frequent social interaction and the easier it is to encourage families to learn financial knowledge. The influence of family income on financial knowledge acquisition was significant, indicating that families with higher income levels have greater demand for financial knowledge.

### 4.3. The mediating effect of knowledge acquisition on urban and rural household financial asset allocation

The estimation results showed that the mediating effect of knowledge acquisition was significant for both urban and rural families, albeit more significant for rural families as a whole. It was found that compared with urban families, rural families with higher collectivism and uncertainty avoidance had fewer high-risk financial assets. Rural family cultural values had a more significant positive impact on household financial asset allocation, and the mediating effect of knowledge acquisition had a more significant impact on rural families. This is because in rural areas with a relatively high degree of uncertainty avoidance, most families hold financial assets with precautionary motives and have little concept of investment and financing. Residents generally belong to a “risk averse” group with a conservative mentality and low level of financial knowledge. A considerable number of rural families are afraid of high-risk financial asset allocation activities. There are also some rural families with considerable income, and most of their surplus funds are saved in banks. These residents have a high frequency of Internet learning and a high desire for financial knowledge. While in urban households with a low uncertainty avoidance degree, residents are more active in new things, investment risks, and long-term orientation, etc. After having surplus funds, residents mostly invest in securities, such as stocks, bonds, funds, and other financial products, but have low demand for financial knowledge acquisition.

The family samples were divided into a high financial asset allocation group and low financial asset allocation group based on the median. Further regression analysis showed that in urban families with high financial asset allocation, family cultural values played a significant mediating role in Internet learning knowledge acquisition, while in rural families with high financial asset allocation, the mediating effect was significant in both educational knowledge acquisition and Internet learning knowledge acquisition. The reason for this difference may be that the overall education level of urban families is relatively high, while that of rural families is relatively low. However, with increasing attention paid to education, rural families with higher education have acquired more financial knowledge and are more willing to allocate financial assets; thus, the mediating effect of rural family knowledge acquisition is more obvious. The regression results of the mediation effect model are shown in Table 4.

**Table 4:** Mediating effect of knowledge acquisition in high households financial asset allocation.

variables	Urban households with high financial asset allocation (*N* = 2,136)			Rural households with high financial asset allocation (*N* = 1,403)		
	Level of education	Frequency of Internet learning	Household financial asset allocation	Level of education	Frequency of Internet learning	Household financial asset allocation
Level of education			0.224*** (2.92)			0.443*** (3.30)
Frequency of internet learning			0.152** (2.29)			0.305*** (2.12)
Family cultural values	0.166 (2.74)	0.372** (2.92)	0.264*** (4.19)	0.288*** (2.62)	0.103** (2.08)	0.265*** (5.19)
Householders’ personal characteristics (control variable)	Yes	Yes	Yes	Yes	Yes	Yes
Family characteristics (control variable)	Yes	Yes	Yes	Yes	Yes	Yes

^*^p < 0.05.

** *p* < 0.01.

*** *p* < 0.001.

## 5. Discussion and conclusion

### 5.1. Discussion

Based on Hofstede’s cultural values and the CFPS survey data, the empirical study in this paper showed that the cultural values of urban and rural families had a significant positive impact on household financial asset allocation. In other words, the higher the financial knowledge level of family members was, the greater the influence of their family financial cultural thoughts and the higher the probability of families choosing financial asset investment. At the same time, the influence of family cultural values on family financial asset allocation through knowledge acquisition was shown to play an intermediary role. Based on the research from the perspective of urban–rural difference, it was found that rural families with higher collectivism and uncertainty avoidance had a more significant positive impact on household financial asset allocation than urban families. Moreover, the mediating effect of knowledge acquisition on financial asset allocation was more significant.

From the perspective of cross-cultural psychology, to achieve the integration of urban and rural culture, both urban and rural residents should make efforts toward it, so as to realize the coexistence and prosperity of both sides. Based on the actual situation of urban and rural areas in China, to promote the realization of common prosperity in urban and rural areas, it is necessary to strengthen the awareness of financial cultures, promote the popularization of financial knowledge, and optimize the financial ecological environment, which will also help relevant departments to understand and formulate beneficial policies, guiding and helping families to make reasonable investments. The following suggestions are thus put forward.

First, we should strengthen our understanding of financial cultures. In the process of developing socialist culture with Chinese characteristics, we should pay attention to the cultivation of financial cultural cognition and improve the financial cultural atmosphere of society as a whole so that residents have adequate financial literacy knowledge, thus cultivating their financial cultural cognition and improving the utilization efficiency of financial information. The government should actively guide residents to be rational in financial asset allocation, neither blindly pursuing high returns while ignoring investment risks nor isolating themselves from the financial market.

Second, we should promote financial literacy. Digital technology should be relied on to expand the channels for residents(Huang et al., 2022), especially rural residents, to obtain financial information and make full use of mobile platforms, Internet, broadcasting, and other channels to carry out financial knowledge publicity, thereby effectively promoting the acquisition of financial knowledge, promoting the accumulation of family financial and cultural capital, and narrowing the gap between urban and rural households in terms of financial asset allocation. The government should increase investment in public financial knowledge products, hold Internet financial knowledge learning classes, explain the business process of handling and purchasing financial products, and enhance the financial literacy of residents. Financial institutions should develop a simplified version of the APP(application) so that rural households with low education levels can conduct financial management conveniently and quickly, and rural residents can have the conditions to allocate household financial assets.

Third, we should improve the financial environment. The government should speed up the development of the financial sector and improve the financial cultural atmosphere in order to penetrate the family unit and continuously optimize the financial ecological environment. For urban areas with strong financial cultures, the government should improve the investment and financial management environment of the financial industry, improve the trust of households in financial market investment, and alleviate and reduce the uncertainty and risk caused by high-risk investment. For the rural areas with relatively late financial market development, the government should coordinate the speed and efficiency of inter-regional financial development and use the advantageous resources of urban financial market development to help rural financial market development.

### 5.2. Limitations and future directions

The contributions of this study mainly lies in the following three aspects: first, it explores the significant positive effects of family cultural psychology and values on household financial asset allocation not only from the perspective of traditional family research but also from the perspective of knowledge acquisition; this further expands the factors affecting household financial asset allocation, as well as enriches the research literature of cultural psychology and household asset allocation. Second, by combining family cultural values with Chinese family tracking survey data, this paper explores the economic impact of family cultural values on household financial asset allocation from the perspective of Hofstede’s values, also empirically verifying the impact of family cultural values on household financial asset allocation. Third, mutual assistance is a traditional virtue of family cultural values advocated by the Chinese nation, which is an important way to promote the acquisition of financial knowledge. Social interactive knowledge learning and acquisition play an important responsible role in actively maintaining social stability and have a positive impact on the construction of harmonious and civilized urban and rural cultural values.

The conclusions of this paper provide a relevant theoretical basis and relevant policy suggestions regarding the impact of promoting the influence of cultural psychology and cultural values in promoting household financial asset allocation. However, the endogeneity of cultural values and household financial asset allocation has not been elaborated, and the difference of multicultural values and the potential impact of knowledge exchange have not been emphasized, which can be further studied through empirical research or case studies or other means in the future.

## Data availability statement

The original contributions presented in the study are included in the article/supplementary material, further inquiries can be directed to the corresponding author.

## Author contributions

ZL is mainly responsible for the introduction and hypotheses part. CM and MD’A write the research methods and empirical results. JJ is the corresponding author and is responsible for the discussion and results section. All authors contributed to the article and approved the submitted version.

## Funding

The paper was supported by the National Social Science Foundation of China (No. 19BJY157), Project of Hunan Social Science Evaluation Committee (No. XSP22YBZZ167) and The Natural Science Foundation of Changsha, China (No. kq2022228).

## Conflict of interest

The authors declare that the research was conducted in the absence of any commercial or financial relationships that could be construed as a potential conflict of interest.

## Publisher’s note

All claims expressed in this article are solely those of the authors and do not necessarily represent those of their affiliated organizations, or those of the publisher, the editors and the reviewers. Any product that may be evaluated in this article, or claim that may be made by its manufacturer, is not guaranteed or endorsed by the publisher.

## Footnotes

^1^Data Source: Global Wealth Report 2022 https://new.qq.com/rain/a/20220927A0285400.

^2^Data source: China Social Science Survey Center, Peking University http://isss.pku.edu.cn/cfps/download/login.
